# Bioactive Materials for Caries Management: A Literature Review

**DOI:** 10.3390/dj11030059

**Published:** 2023-02-23

**Authors:** Olivia Lili Zhang, John Yun Niu, Iris Xiaoxue Yin, Ollie Yiru Yu, May Lei Mei, Chun Hung Chu

**Affiliations:** 1 Faculty of Dentistry, The University of Hong Kong, Hong Kong, China; 2 Faculty of Dentistry, The University of Otago, Dunedin 9054, New Zealand

**Keywords:** antimicrobial, remineralisation, caries, peptides, prevention, bioactive, dental materials

## Abstract

Researchers have developed novel bioactive materials for caries management. Many clinicians also favour these materials, which fit their contemporary practice philosophy of using the medical model of caries management and minimally invasive dentistry. Although there is no consensus on the definition of bioactive materials, bioactive materials in cariology are generally considered to be those that can form hydroxyapatite crystals on the tooth surface. Common bioactive materials include fluoride-based materials, calcium- and phosphate-based materials, graphene-based materials, metal and metal-oxide nanomaterials and peptide-based materials. Silver diamine fluoride (SDF) is a fluoride-based material containing silver; silver is antibacterial and fluoride promotes remineralisation. Casein phosphopeptide-amorphous calcium phosphate is a calcium- and phosphate-based material that can be added to toothpaste and chewing gum for caries prevention. Researchers use graphene-based materials and metal or metal-oxide nanomaterials as anticaries agents. Graphene-based materials, such as graphene oxide-silver, have antibacterial and mineralising properties. Metal and metal-oxide nanomaterials, such as silver and copper oxide, are antimicrobial. Incorporating mineralising materials could introduce remineralising properties to metallic nanoparticles. Researchers have also developed antimicrobial peptides with mineralising properties for caries prevention. The purpose of this literature review is to provide an overview of current bioactive materials for caries management.

## 1. Introduction

Dental caries is challenging to human health and society. It is the most common and worldwide biofilm-dependent disease [1], where nearly half of the population worldwide suffers from untreated caries [2,3]. The situation of high prevalence of early childhood caries could be improved with positive socio-behavioural factors [4]. Even though dental health care professionals have made significant efforts to control dental caries, the incidence rate over the past few years has remained high [5]. Caries prevalence has decreased only 4% from its level 30 years ago [6]. Dental caries result from demineralisation and remineralisation cycles at the interface of biofilm and tooth surface [7]. The demineralisation is caused by acid produced by oral biofilm. The new philosophy of caries management is minimal intervention, which aims to maintain the dental structure and pulpal vitality to prolong the tooth’s life [8]. Minimal intervention highlights the “minimal” or “nonrestorative” techniques to control caries by inhibiting mineral loss [9]. However, the most common approach for caries management remains traditional drilling and filling [10], from which it is necessary to shift to a new concept: a medical model [11]. In addition, conditions such as white spot lesions and molar incisor hypomineralisation should also be considered precursors of dental caries [12,13]. These conditions should be managed in clinical settings using bioactive materials [12].

Therefore, researchers have developed novel bioactive materials for caries management [14]. In medicine, a “biomaterial” is an artificial substance that interacts with components of living systems [15]. The biomaterial can be used in therapeutic or diagnostic medical procedures alone or as part of a complex system [15]. Alongside the development of biomaterials, the concept of “bioactive” materials has emerged. Bioactive materials elicit special chemical bonding at the interface between tissues and the material [16]. For example, fluoride-releasing materials are typical bioactive restorative materials in dentistry. They have been widely applied in clinical settings to prevent secondary caries because the fluoride-releasing material adheres to the tooth tissue and releases fluoride [17].

There is no consensus on the definition of bioactive materials. The term “bioactive” applies to a dental material and should describe a beneficial biological process in which the material actively stimulates specific tissue or cellular responses or interacts with pathogenic species [16]. Therefore, bioactive materials in cariology/restorative dentistry generally are considered materials capable of forming hydroxyapatite crystals on tooth surfaces. In addition, dental caries is a non-communicable, chronic and behaviour-mediated biofilm disease [18]. The biofilm form develops through a multi-stage process, including bacterial attachment, microcolony formation, biofilm maturation and biofilm dispersal [19]. A bioactive material intended to control caries should be able to disrupt any or all stages of biofilm formation.

In recent years, more bioactive materials with mineralising and/or antibacterial properties for caries treatment have emerged [14] with the development of nanotechnology [20]. Many clinicians also favour these materials that fit their contemporary practice philosophy of the medical model of caries management and minimally invasive dentistry [11]. Common bioactive materials for caries management include fluoride-based materials, calcium- and phosphate-based materials, graphene-based materials, metal and metal-oxide nanomaterials and peptide-based materials (Table 1).

For example, silver diamine fluoride (SDF) can effectively prevent and arrest enamel and dentine caries [39,40]. In addition, peptides with antimicrobial and mineralising properties can inhibit cariogenic biofilm and promote remineralisation [36,38]. Ideal bioactive materials should be biocompatible, stable, non-discolouring and low cost [41]. Thus far, no previous review article describes the developing status of bioactive materials for caries management. The aim of this literature review is to provide an overview of current bioactive materials for caries management.

## 2. Fluoride-Based Materials

Fluoride is essential for the prevention of dental caries [21]. The World Health Organization passed a resolution stating that universal access to fluoride for caries prevention is a fundamental human right [42]. Water fluoridation is integral to oral health programs worldwide [43]. Fluoride inhibits demineralisation and enhances remineralisation of the teeth’s hard tissue. Topical application of fluorides influences tooth surfaces. After fluoride therapy, a layer of calcium fluoride forms, which can be incorporated into the fluorapatite of the tooth surface [44]. All of these increase tooth minerals’ acid resistance, or in other words, reduce the critical pH value of mineral dissolution. In addition, fluoride can play a role in inhibiting cariogenic biofilm [44]. 

Various formulations of fluoride-based materials in various fluoride concentrations are applied in dentistry. The most common formulations include SDF, sodium fluoride, sodium monofluorophosphate, acidulated fluorophosphate, stannous fluoride and amine fluoride. These fluoride-based materials can be delivered in various forms, including mouth rinses, toothpastes, foams, gels and varnishes [41]. The fluoride concentration, type of fluoride compound and frequency and duration of application significantly affect topical fluoride applications’ efficacy in caries prevention [45]. 

The World Health Organization recommends the daily use of low-concentration fluorides in toothpaste, and it is believed that everyday fluoride toothpaste use is the main reason for the reduction of caries prevalence in past decades [46]. In addition, professional fluoride therapy to arrest active dental caries is an effective, low-cost and easily operated strategy [41]. 

### 2.1. Silver Diamine Fluoride

SDF was invented in the 1960s in Japan [47]. It is a fluoride-based material containing silver, fluoride and ammonia, with a pH value between 9 and 10. Silver is an effective antibacterial agent, and fluoride is an effective remineralisation agent. The most common concentration of SDF solution is 38% (44,800 ppm of fluoride), but 30% (35,400 ppm) and 12% (14,150 ppm) SDF solutions are also used [48]. Research has demonstrated that 12% SDF is less effective in arresting caries than more concentrated solutions [49,50]. In comparison, a 38% SDF solution can arrest 81% of dentine caries in primary dentition [51]. In addition, 38% SDF acts more quickly to arrest caries than 5% fluoride varnish does [52] and arrests root caries effectively [53]. SDF’s advantages are that it is easy to operate, non-invasive, painless, non-aerosol-generating and inexpensive [47]. 

The World Health Organization recognised SDF as an important medicine [54]. SDF therapy is effective for caries arrest and prevention, especially for difficult-to-treat lesions and elderly patients or patients with difficulties in routine hygiene procedures [55,56]. However, it still induces some side effects, such as black staining and a metallic taste, which may cause dissatisfaction among children and parents [57]. Therefore, further research on the use of SDF should focus on mitigating these disadvantages.

### 2.2. Sodium Fluoride

Sodium fluoride is also one of the most-used fluoride materials. It can exist in the forms of 0.05% and 0.2% rinses, 2% gel and 5% varnish. As early as 1964, a varnish containing sodium fluoride in a natural colophony base was invented [58]. Sodium fluoride varnish is a slow-releasing reservoir that helps fluoride adhere to and stay on tooth surfaces in the oral environment [59]. Today, 5% (containing 22,600 ppm fluoride) sodium fluoride varnish is suggested to be biannually applied in children and teenagers to prevent dental caries [60]. Serial studies have demonstrated that sodium fluoride varnish is safe [61] and cost-effective [59]. It can effectively reduce early childhood caries development in children [62,63]. In addition, sodium fluoride varnish effectively reverses initial caries and early enamel lesions [22,51]. However, the effect on arresting cavitated caries of sodium fluoride varnish is not ideal [64].

### 2.3. Other Fluoride-Based Materials

Other common fluoride-based materials include sodium monofluorophosphate, acidulated fluorophosphate, stannous fluoride and amine fluoride. Sodium monofluorophosphate is an active anticaries agent in toothpaste [23]. Toothpaste containing sodium monofluorophosphate showed efficacy similar to that of toothpaste containing sodium fluoride [23]. Acidulated fluorophosphate gel is the most commonly used professional topically applied fluoride gel [46,59]. However, topical application of acidulated fluorophosphate gel has no additional arresting effect on caries lesions [24]. Stannous fluoride has more antimicrobial properties than other fluoride compounds do [65,66]. Therefore, it has been widely used in many oral care products. However, stannous fluoride’s stability is not ideal, and it may cause dark staining [25]. Amine fluoride is available in toothpastes, gels and mouth rinses [26]. It can reduce plaque adhesion and remain longer in the oral cavity because of its strong affinity with the enamel surface [25,67]. However, research on sodium monofluorophosphate, acidulated fluorophosphate, stannous fluoride and amine fluoride is insufficient, and their action mechanisms are still unclear. It is necessary to carry out more studies to confirm their clinical effectiveness.

## 3. Calcium- and Phosphate-Based Materials

The conception of “remineralisation” in cariology refers to mineral gain on the demineralised enamel and dentine surface [68]. The source of calcium and phosphate could be saliva or external sources. The saliva can help remineralise demineralised enamel crystals, but it is insufficient for tissue repair because saliva tends only to remineralise a lesion’s surface layer. The reason for this limited scope of remineralisation is that the ion concentration gradient from the saliva into the lesions is low. Therefore, introducing various calcium- and phosphate-based agents to provide abundant calcium and phosphate sources is a strategy of caries management. These kinds of materials include casein phosphopeptide-amorphous calcium phosphate (CPP-ACP), casein phosphopeptide-amorphous calcium fluoride phosphate (CPP-ACFP), functionalised β-tricalcium phosphate, nano-hydroxyapatite and calcium sodium phosphosilicate [69,70].

### 3.1. CPP-ACP and CPP-ACFP

CPP-ACP comprises nanoclusters of casein phosphopeptide bound to amorphous calcium phosphate [27]. CPP stabilises calcium and phosphate ions by forming CPP-ACP complexes [71]. CPP-ACP promotes the remineralisation of subsurface lesions by supplying a high concentration of calcium and phosphate ions [68,72,73,74]. CPP-ACP products include chewing gums, mouthwashes and dental creams [68]. These products exhibit anticaries properties without unpalatability or allergenicity [75]. A 24-month clinical trial showed that chewing a CPP-ACP gum significantly reduced the progress of caries [72]. A 6-month clinical trial showed that a toothpaste containing CPP-ACP had a better reduction effect on *Streptococcus mutans*-positive children than did fluoride toothpaste alone or combined with both agents [76]. Some other studies showed that using 10% CPP-ACP cream over 12 months [77] and 24 months [78] can significantly reduce the number of *Streptococcus mutans*-positive children. However, it cannot significantly reduce caries prevalence. Comprehensive evidence has confirmed that although CPP-ACP has a remineralising property, it does not differ significantly from that of fluoride products [79]. Therefore, CPP-ACP can be used as an adjunct, not an alternative to fluorides [80]. 

CPP-ACPF came into being through the addition of 0.2% fluoride (900 ppm) to CPP-ACP [28]. CPP-ACPF has shown an increased remineralising effect thanks to the distribution of fluoride ions, calcium ions and phosphate ions, which enables substantial crystal growth and fluorapatite formation [28,74,81]. An in vitro study showed that combining CPP-ACFP with conventional toothpaste on initial caries lesions increased enamel’s mineral content after 6 weeks and 12 weeks [82]. However, systemic evidence showed that CPP-ACPF is less effective than CPP-ACP alone [83]. It is necessary to design and conduct high-quality clinical trials to confirm the efficacy of CPP-ACP and CPP-ACFP for dental caries management.

### 3.2. Functionalised β-Tricalcium Phosphate

Another calcium phosphate system, β-tricalcium phosphate (β-TCP), is widely investigated in bone remodelling and orthopaedic applications [29]. β-TCP allows for crystal modification [29]. Therefore, researchers are trying to modify β-TCP to make it compatible with fluoride [21]. Functionalised β-TCP (fTCP) can create barriers to prevent interactions between fluoride and calcium. In addition, fTCP can target delivery of minerals and fluoride to the teeth’s surface [84]. The minerals produced by the combination of fluoride and fTCP showed greater acid resistance than that of fluoride, β-TCP and fTCP [84,85]. However, the clinical evidence remains insufficient. Further research is needed to assess fTCP’s effectiveness in caries remineralisation [70].

### 3.3. Nano-Hydroxyapatite Materials

Hydroxyapatite is the tooth’s main component of hard tissue [86]. Nano-hydroxyapatite materials can be used for dental implant coating, desensitisation and remineralisation [87,88]. Nano-hydroxyapatite can directly fill and cover the demineralised enamel due to its strong affinity with enamel [30]. The filled nano-hydroxyapatite is the scaffold of remineralisation. It facilitates the gathering of calcium and phosphate from saliva to the enamel surface, forming a new apatite layer [30]. Even in acidic conditions, nano-hydroxyapatite still significantly increases remineralisation by attracting mineral ions into the demineralised zone [89]. In addition, nano-hydroxyapatite can increase hard tissue’s micro-hardness and decelerate caries’ development [90,91]. Several in vitro studies have found that nano-hydroxyapatite toothpaste can remineralise the artificial initial caries [92,93]. Moreover, nano-hydroxyapatite showed a better remineralising effect than fluoride varnish did [27]. However, the clinical evidence is still insufficient to support conclusions about the remineralising effect of nano-hydroxyapatite on dental caries [94].

### 3.4. Calcium Sodium Phosphosilicate

Calcium sodium phosphosilicate is a kind of bioactive glass which is another potential anticaries agent [31]. It is a stable composite of SiO_2_, CaO and Na_2_O. It has ideal biocompatibility without toxicity, inflammation or foreign-body response [31]. Calcium sodium phosphosilicate is dissolvable and can release sodium, calcium and phosphate in saliva. This is essential for generating a hydroxyapatite layer on the demineralised tooth surface [95,96]. Laboratory studies have demonstrated that calcium sodium phosphosilicate promotes remineralisation and inhibits demineralisation on enamel and dentine [97,98,99]. In addition, calcium sodium phosphosilicate also has broad-spectrum antimicrobial properties [100]. Although the FDA has approved calcium sodium phosphosilicate (Bioglass^®^ 45S5) for clinical applications, the evidence of bioactive glass’s clinical efficacy against caries is still insufficient [101].

Even though a series of calcium- and phosphate-based materials showed remineralising ability [102], no clinical evidence has shown that calcium- and phosphate-based materials could replace the common use of fluoride in the minimal intervention treatment for caries [103]. Calcium- and phosphate-based materials can be used to supplement fluoride treatments because the calcium and phosphate ions are necessary for forming fluorapatite [28]. In addition, in situ enamel remineralisation and fluoride retention in plaque depend on the availability of calcium ions [104].

## 4. Graphene-Based Materials

Graphene is a single flat sheet of carbon-based nanomaterial with an ideal two-dimensional (2D) structure [32]. Unfortunately, it is not easy to synthesise large-scale graphene. Therefore, derivatives of graphene, such as graphene oxide (GO) and reduced graphene oxide (rGO), have been introduced because they are easier to produce [105]. Graphene and its derivatives have many applications in science and technology, including dentistry [32,106]. For caries management, graphene-based materials have anti-cariogenic pathogen capacities [107] and mineralising properties [108]. GO has active sites which allow for its functional combination with other bioactive molecules [109] whereas rGO is made by eliminating the oxygen in GO to induce graphene-like behaviour [110]. 

Researchers have found that GO can be combined with other materials to reveal its anticaries ability in vitro [105]. Nizami et al. found that GO-silver (Ag) and GO-Ag-calcium fluoride (GO-Ag-CaF2) prevented the demineralisation of dentine and inhibited the growth of *Streptococcus mutans* [108]. Wu et al. combined GO with Antisense vicR RNA, demonstrating its ability to reduce bacterial growth and suppress biofilm formation [111]. Khosalim et al. observed that GO can foster hydroxyapatite and improve the nano-hardness of dentine slices [112]. Zanni et al. indicated that zinc oxide nanorod-decorated graphene nanoplatelets inhibit the formation of *Streptococcus mutans* biofilm [113]. Mao et al. found that graphene oxide-copper nanocomposites could hinder the growth of *Streptococcus mutans* [114]. 

However, researchers still need to discuss graphene-based materials’ biocompatibility and toxicity comprehensively [106]. Translation of graphene-based materials for clinical application is still distant.

## 5. Metal and Metal-Oxide Nanomaterials

Because nanotechnology offers a powerful defence against microbial proliferation, researchers have developed anticaries nanomaterials [34]. Various metal and metal-oxides, such as silver, zinc oxide and copper oxide, have antimicrobial abilities [34]. The antimicrobial mechanism of metallic nanoparticles includes metal ion release and oxidative stress induction. In addition, the nanoscale particle sizes increase the surface-to-volume ratio, which facilitates materials’ damage to the extracellular polysaccharide bacterial matrix and the penetration of bacterial membranes [115]. Moreover, incorporating mineralising materials could introduce remineralising properties to metallic nanoparticles [116].

### 5.1. Silver Nanoparticles

Silver nanoparticles (AgNPs) have been widely studied [117]. AgNPs can inhibit the growth and adhesion of cariogenic pathogens, especially *Streptococcus mutans* [33]. AgNPs also have antiviral and antifungal qualities. Studies have demonstrated AgNPs’ broad-spectrum antimicrobial properties in vitro [118]. Anticaries agent-based AgNPs have a practical bactericidal effect against *Streptococcus mutans* biofilm [119]. In addition, AgNPs can slow the demineralisation of teeth by inhibiting lactic acid production and collagen degradation by biofilm [120].

To add mineralising properties to AgNPs, researchers have tried to combine AgNPs with fluoride [119]. An in vitro study showed that the combination of fluoride sodium with AgNPs showed greater remineralisation efficacy than sodium fluoride alone and nano-hydroxyapatite on enamel [121]. However, another in vitro study showed that toothpaste containing AgNPs and fluoride sodium affected the demineralised enamel, such as sodium fluoride toothpaste [122]. In addition, Yin et al. found no significant difference between AgNPs with sodium fluoride and sodium fluoride alone in terms of the remineralising effect [33].

Nano-silver fluoride is an AgNP product comprising chitosan and fluoride. It has long-term stability and good biocompatibility and does not stain the enamel [123]. A randomised clinical trial showed that the yearly application of nano-silver fluoride arrested 66.7% of dentine caries in primary teeth at 12 months [124]. Another clinical trial showed that annual nano-silver fluoride application prevented dentine caries’ progression in primary molars [125]. In addition, the combination of AgNPs and fluoride sodium or nano-silver fluoride does not cause black staining and metallic taste, unlike SDF [120,126]. Apparently, the AgNPs’ properties suggest their potential as an effective anticaries agent without black staining. However, nanoparticles’ toxicity cannot be ignored. Studies have shown that toxicity depends on the concentration and types of silver nanoparticles [127]. Therefore, well-designed clinical trials are essential to prove further AgNPs’ effectiveness for clinical application.

### 5.2. Zinc Oxide Nanoparticles

Zinc oxide nanoparticles have better antimicrobial properties and biocompatibility than zinc nanoparticles do [34,128]. Researchers have found that incorporating zinc oxide nanoparticles gives adhesives antimicrobial properties [129]. For example, Kasraei et al. found that composite resins containing zinc oxide or silver nanoparticles showed antimicrobial activity against *Streptococcus mutans* [130]. Swetha et al. found that pit and fissure sealants containing zinc oxide nanoparticles also showed antimicrobial activity [131].

### 5.3. Copper Nanoparticles

Copper nanoparticles also have shown effectiveness against cariogenic bacteria [35,132]. In addition, copper nanoparticles can achieve a better cost–benefit ratio than that of silver nanoparticles [34]. Covarrubias et al. synthesised copper chitosan hybrid nanoparticles with antimicrobial activities against *Streptococcus mutans* [133]. Altankhishig et al. demonstrated that incorporating zinc and copper nanocomposite gave the adhesive system antibacterial properties [134].

Metal and metal-oxide nanomaterials are antimicrobial. They can be combined with mineralising materials to enhance their anticaries properties. However, further research is necessary to translate nanomaterials for dental caries management into clinical settings.

## 6. Peptide-Based Materials

Peptides are usually strings of 2 to 50 amino acids [36]. Researchers seek peptides with antimicrobial and mineralising effects [135]. A series of datasets on peptides for caries management has been developed [136]. Some natural peptides are chemically modified to improve their activity and biocompatibility [137]. In addition, the shorter the peptide, the lower its production cost [138].

### 6.1. Antimicrobial Peptides

Antimicrobial peptides have attracted researchers’ attention [36]. Natural antimicrobial peptides in oral cavities are the first line of defence against oral pathogenic microbes, such as *Streptococcus mutans* and *Candida albicans* [139,140]. Due to their promising antimicrobial abilities and low likelihood of inducing resistance, an increasing number of synthetic artificial antimicrobial peptides have been introduced [136]. Artificial antimicrobial peptides can be developed by modelling natural antimicrobial peptides, new designs and functional modifications. For example, KR12-KAKE is a novel antimicrobial peptide derived from LL37, which can inhibit the growth of *Streptococcus mutans* biofilm [141]. GH12, a newly designed antimicrobial peptide, shows antimicrobial activity against cariogenic pathogens and biofilms in vitro [142]. In addition, the peptide C16G2, modified from the broad-spectrum antimicrobial peptide G2, specifically targets *Streptococcus mutans* [143].

### 6.2. Mineralising Peptides

Peptides with mineralising effects, such as self-assembling peptides, have been proven in vitro as an effective strategy for treating initial caries [37]. For example, P_11-4_ is a self-assembling peptide which can form a 3D scaffold in carious lesions and promote the new formation of hydroxyapatite crystal, in what is called guided enamel regeneration [144]. Cao et al. developed a novel oligopeptide by fusing the hydrophilic C-terminal of amelogenin and the collagen-binding domain of DMP1 [145]. This novel oligopeptide can induce dentine’s remineralisation by binding collagen fibrils [145].

### 6.3. Peptides with Mineralising and Antimicrobial Properties

Researchers are also developing peptides with mineralising and antimicrobial properties targeted at caries management. Niu et al. developed a novel peptide, GA-KR12, which has broad-spectrum antimicrobial properties against cariogenic bacteria and *Candida albicans* [38]. This peptide can also remineralise initial enamel and dentine caries [146,147]. Wang et al. developed a peptide that can inhibit *Streptococcus mutans* and induce remineralisation [148]. Although many studies have focused on applying peptide-based materials for caries management, these materials are still in the early stages of laboratory studies [149]. Researchers should focus on promoting the translation to clinical use of peptides for caries management.

## 7. Prospect of Bioactive Materials for Caries Management

Because dental caries is a prevalent oral disease and bioactive materials have desirable remineralising properties, researchers are interested in developing novel bioactive materials for caries management. Table 2 shows the commercialisation status of bioactive materials mentioned in this paper.

Some of the synthesised bioactive materials including fluoride-based materials, calcium- and phosphate-based materials and peptide-based materials have been commercially produced. Topical fluoride products such as toothpastes, fluoride gels, fluoride rinse and fluoride varnishes can be used at-home or in-office [150]. Calcium- and phosphate-based materials are used as at-home dental care products. At present, only one peptide-based material is commercially available has for clinical use. Although researchers developed a variety of bioactive materials with promising remineralising properties, some of the materials are not yet commercialised. The researchers demonstrated that graphene-based materials, metal and metal-oxide nanomaterials and peptide-based materials are effective to remineralising demineralised lesions [32,33,36,38,151,152]. They are, however, mostly laboratory studies. Only a few are pre-clinical trials, and hence, the clinical evidence is not strong. Basic research is the foundation of translational research [153,154]. In the near future, there will be translation research to promote the application of novel bioactive materials for caries management. Conventional caries management is a surgical approach to replace the carious tissue with restoration [11]. More and more clinicians are now managing dental caries using minimal invasive approach and medical model [69].

## 8. Conclusions

In conclusion, studies have demonstrated that bioactive materials, including fluoride-based materials, calcium- and phosphate-based materials, graphene-based materials, metal and metal-oxide nanomaterials and peptide-based materials, possess ideal antimicrobial and remineralising properties for managing dental caries; however, the clinical evidence is still insufficient. Therefore, further research, accordingly, is essential to promote the clinical application of novel bioactive materials for caries management.

## Figures and Tables

**Table 1:** Desirable properties of various bioactive materials.

Bioactive Materials	Desirable Property (● Yes ● Inconclusive ● No)				
	Biocompatible	Antibacterial	Stable	Nondiscolouring	Low-Cost
***Fluoride-based materials***					
Silver diamine fluoride [21]	●	●	●	●	●
Sodium fluoride [22]	●	●	●	●	●
Sodium monofluorophosphate [23]	●	●	●	●	●
Acidulated fluorophosphate [24]	●	●	●	●	●
Stannous fluoride [25]	●	●	●	●	●
Amine fluoride [26]	●	●	●	●	●
***Calcium- and phosphate-based materials***					
Casein phosphopeptide-amorphous calcium phosphate [27]	●	●	●	●	●
Casein phosphopeptide-amorphous calcium fluoride phosphate [28]	●	●	●	●	●
Functionalised tricalcium phosphate [29]	●	●	●	●	●
Nano-hydroxyapatite [30]	●	●	●	●	●
Calcium sodium phosphosilicate [31]	●	●	●	●	●
***Graphene-based materials***					
Graphene [32]	●	●	●	●	●
Graphene oxide and reduced graphene oxide [32]	●	●	●	●	●
***Metal and metal-oxide nanomaterials***					
Silver nanoparticles [33]	●	●	●	●	●
Zinc oxide nanoparticles [34]	●	●	●	●	●
Copper nanoparticles [35]	●	●	●	●	●
***Peptide-based materials***					
Antimicrobial peptides [36]	●	●	●	●	●
Mineralising antimicrobial peptides [37]	●	●	●	●	●
Mineralising peptides [38]	●	●	●	●	●

**Table 2:** Some commercial products of bioactive materials.

Bioactive Materials	Commercial Product, Brand	Use
***Fluoride-based materials***		
Acidulated fluorophosphate	Gelato APF Gel, Keystone Industries	In-office
Silver diamine fluoride	38% SDF, Advantage Arrest	In-office
Sodium fluoride	Duraphat Varnish, Colgate	In-office
Amine fluoride	Active Remineralization Toothpaste, Amflor	At-home
Sodium monofluorophosphate	Anticavity Toothpaste, Colgate	At-home
Stannous fluoride	PerioMed Oral Rinse, 3M	At-home
***Calcium- and phosphate-based materials***		
Calcium sodium phosphosilicate	NovaMin toothpaste, Sensodyne	At-home
Casein phosphopeptide-amorphous calcium phosphate	Tooth Mousse, GC Europe	At-home
Casein phosphopeptide-amorphous calcium fluoride phosphate	Tooth Mousse Plus, GC Europe	At-home
Functionalised tricalcium phosphate	Clinpro™ 5000 Toothpaste, 3M	At-home
Nano-hydroxyapatite	Pro-Mineralizer Toothpaste, Great Oral Health	At-home
***Graphene-based materials***		
Graphene	No Commercial product available	-
Graphene oxide and reduced graphene oxide	No Commercial product available	-
***Metal and metal-oxide nanomaterials***		
Silver nanoparticles	No Commercial product available	-
Zinc oxide nanoparticles	No Commercial product available	-
Copper nanoparticles	No Commercial product available	-
***Peptide-based materials***		
Mineralising peptides	Curodont Repair, Straumann	In-office
Antimicrobial peptides	No Commercial product available	-
Mineralising antimicrobial peptides	No Commercial product available	-

## Data Availability

Data is contained within the article.
